# CREB Is Activated by Muscle Injury and Promotes Muscle Regeneration

**DOI:** 10.1371/journal.pone.0024714

**Published:** 2011-09-13

**Authors:** Randi Stewart, Lawrence Flechner, Marc Montminy, Rebecca Berdeaux

**Affiliations:** 1 Department of Integrative Biology and Pharmacology, University of Texas Health Science Center at Houston, Houston, Texas, United States of America; 2 Clayton Foundation Laboratory for Peptide Biology, The Salk Institute for Biological Studies, San Diego, California, United States of America; Institute of Genetics and Molecular and Cellular Biology, France

## Abstract

The cAMP response element binding protein (CREB) plays key roles in differentiation of embryonic skeletal muscle progenitors and survival of adult skeletal muscle. However, little is known about the physiologic signals that activate CREB in normal muscle. Here we show that CREB phosphorylation and target genes are induced after acute muscle injury and during regeneration due to genetic mutation. Activated CREB localizes to both myogenic precursor cells and newly regenerating myofibers within regenerating areas. Moreover, we found that signals from damaged skeletal muscle tissue induce CREB phosphorylation and target gene expression in primary mouse myoblasts. An activated CREB mutant (CREBY134F) potentiates myoblast proliferation as well as expression of early myogenic transcription factors in cultured primary myocytes. Consistently, activated CREB-YF promotes myoblast proliferation after acute muscle injury *in vivo* and enhances muscle regeneration in dystrophic *mdx* mice. Our findings reveal a new physiologic function for CREB in contributing to skeletal muscle regeneration.

## Introduction

Vertebrate myogenesis is controlled by cascades of muscle-specific transcription factors, which dictate myogenic specification and differentiation, as well as repair of damaged adult skeletal muscle [1]. The second messenger cAMP and the cAMP-responsive transcription factor CREB are temporally regulated during myogenesis and required for somite development in mouse embryos [2], [3], [4]. Agents that induce cAMP signaling improve muscle strength in humans and mice with muscle disease [5], but little is known about how cAMP-dependent transcription in myogenic precursor cells may contribute to regeneration of damaged adult muscle.

Numerous extracellular signals including those that increase cAMP induce CREB phosphorylation on a conserved serine residue (Ser133) that is required for recruitment of the related histone acetyltransferases CBP/p300 [reviewed in 6]. Although it is currently unknown what signals induce CREB(S133) phosphorylation in myoblasts within adult skeletal muscle, genetic studies in mice have shown that CREB activity is required for muscle development and survival. Genetic deletion of *Creb* or expression of a dominant CREB inhibitor termed A-CREB impairs myotome development in mice, possibly via regulation of the myogenic regulators *Pax3* and *Myf5* [2]. Additionally, transgenic expression of A-CREB in mature myofibers causes muscle degeneration [7]. CREB promotes survival of differentiated muscle by transcriptional induction of the target gene salt inducible kinase 1 (*Sik1*), which couples CREB and MEF2 transcription by direct phosphorylation and inhibition of class II histone deacetylases [7]. CREB has also been shown to regulate *RB* [8] and *follistatin* [9] transcription during myogenic differentiation, suggesting that CREB is involved in terminal cell cycle arrest and fusion during myogenesis. Together these findings show that CREB is an important regulator of multiple stages of muscle differentiation and survival, likely via distinct sets of target genes.

Myogenic differentiation not only occurs during muscle development, but also during muscle regeneration [1]. Upon acute muscle injury or damage due to genetic mutations, resident muscle stem cells, or satellite cells, become activated, proliferate, migrate to the site of damage and fuse with each other and existing myofibers to restore muscle structure. Injured muscle releases numerous signaling molecules including growth factors (HGF, FGFs, PDGF), Wnts, TGF-beta family ligands, and G-protein coupled receptor ligands [1], [10]. These signals promote regeneration in part by activating quiescent satellite cells and providing homing cues for migrating myoblasts and macrophages. As many of these signals activate CREB [6] and CREB activity is required for myogenic differentiation during embryogenesis [2], CREB is ideally situated to mediate regenerative responses to signals released in damaged skeletal muscle. However, it is still unknown whether CREB activity is dynamically regulated in myoblasts after muscle injury and how CREB contributes to muscle regeneration. Mouse models with persistent CREB inhibition do not permit analysis of CREB action in this dynamic setting, so we tested the hypothesis that CREB activation contributes to regeneration using primary mouse myoblasts and knock-in mice expressing activated CREB. We show that CREB phosphorylation and target genes are activated in response to skeletal muscle injury and that activated CREB drives myoblast proliferation. Moreover, genetic activation of CREB promotes proliferation after acute muscle damage and regeneration in mice with muscular dystrophy. Our data support a model in which CREB promotes satellite cell proliferation and skeletal muscle regeneration after muscle injury.

## Results and Discussion

### CREB is activated in response to muscle damage

**Figure 1:**
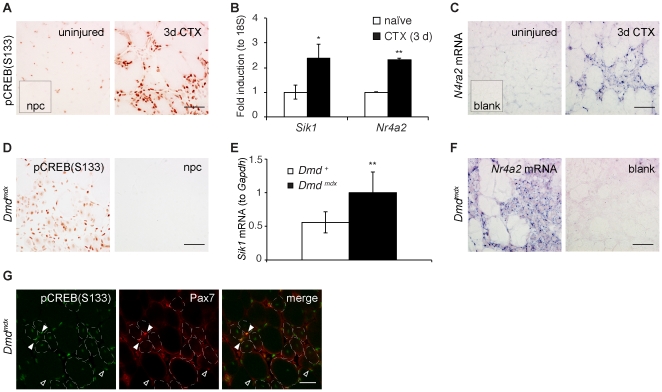
CREB is activated by muscle injury. A) Immunohistochemistry with anti-pCREB(S133) antibody shown on uninjured and injured contralateral muscles 3 days after cardiotoxin (CTX) injection; inset, no primary antibody control (npc). B) CREB target gene mRNAs *Sik1* and *Nr4a2* in mouse gastrocnemius muscle 3 days after CTX injection. Relative mRNA amounts to 18 S rRNA, normalized to naïve muscle. *n = *2 naïve, 3 CTX. * *p*<.05, ** *p*<.01. Similar data were obtained on an independent cohort. C) *In situ* hybridization with *Nr4a2* antisense riboprobe on uninjured and 3 day CTX-injected contralateral legs; no riboprobe control (blank, inset). D) Phospho-CREB immunohistochemistry and npc on gastrocnemius muscle from a 4-week old *Dmd^mdx^* mouse. E) *Sik1* mRNA in wild-type and *Dmd^mdx^* gastrocnemius tissue from 4-week old mice. mRNA normalized to *Gapdh*, averaged by genotype (±stdev); *n = *6 WT, 6 *Dmd^mdx^*; ** *p*<.01. F) *In situ* hybridization with antisense *Nr4a2* riboprobe or blank (control) on *Dmd^mdx^* gastrocnemius at 4 weeks of age. G) Immunofluorescence staining of *Dmd^mdx^* gastrocnemius (4 wk) showing pCREB (green), Pax7 (red) and merge. Filled arrowheads, co-localized Pax7 and pCREB; open arrowheads, Pax7-positive nuclei without high pCREB; dotted circles, centrally nucleated myofibers. Bars, 50 µm (A, C, D, F), 10 µm (G).

To characterize the role of CREB in skeletal muscle regeneration, we injected the snake venom component cardiotoxin into mouse gastrocnemius muscles. Cardiotoxin is commonly used to induce muscle regeneration in experimental models [1]. After cardiotoxin injection, skeletal muscle degeneration and regeneration occur by a well-characterized process of myofiber necrosis, satellite cell activation and differentiation, myoblast proliferation and migration, and eventual myofiber regeneration [11]. Within three days of cardiotoxin injury, we observed striking induction of phosphorylated CREB(Ser133) in the injured areas (Figure 1A and Figure S1A,B), which are evident as large regions of mononucleate cells. These regions are comprised of proliferating myoblasts [11] and infiltrating immune cells [12]. To monitor CREB transcriptional activity, we quantified mRNAs of two direct CREB target genes in muscle cells, *Sik1* and *Nr4a2* [7]. Both of these genes have been previously shown to contain consensus CREB binding sites that are occupied by CREB and phospho-CREB in multiple cell types [13], [14], [15], including C2C12 myoblasts [7]. Moreover, acute induction of these genes is blocked in myoblasts and other cell types by the dominant CREB inhibitor A-CREB [7]. In skeletal muscle, *Sik1* mediates CREB-dependent myofiber survival [7]. Although *Nr4a2* is induced by adrenergic agonists in muscle cells [reviewed in 16], the role of this orphan nuclear receptor in muscle physiology is unknown. Consistent with our results showing phosphorylated CREB in injured skeletal muscle tissue, we found that cardiotoxin treatment induced *Sik1* and *Nr4a2* mRNAs, which peaked 3 days post-injury (Figure 1B and not shown). This time point corresponds to the period of rapid myoblast proliferation [17].

Cardiotoxin injection into the gastrocnemius muscle causes local skeletal muscle degeneration and regeneration, leaving a substantial amount of the muscle tissue intact (not shown). To determine whether CREB target gene mRNA induction was localized to the damaged region of the skeletal muscle, we performed *in situ* hybridization with an antisense probe to the *Nr4a2* transcript [18]. We found that *Nr4a2* expression was strongly localized to the injured region (Figure 1C), similar to phospho-CREB. Control serial sections incubated with no riboprobe showed no staining (Figure 1C, inset). We confirmed that the probe matched the previously reported pattern of *Nr4a2* expression in P14.5 mouse embryos [not shown and 18].

We next investigated whether CREB activation is a general feature of regenerating skeletal muscle. Several genetic mutations in mice and humans result in skeletal muscle degeneration and regeneration; many are clinically relevant, such as the missense mutation in the *Dystrophin* gene that underlies Duchenne's muscular dystrophy [19], [20]. We therefore tested CREB activity in skeletal muscle of dystrophin-deficient *Dmd^mdx^* mice, a genetic model of muscular dystrophy in which a “crisis” of myofiber degeneration and regeneration occurs at 4 weeks of age [21]. Although animals at this age invariably show dramatic muscle degeneration and regeneration, the tissue architecture is heterogeneous within a given muscle. Some regions are highly infiltrated with inflammatory cells and regenerating, while others appear histologically normal. Similar to our findings in cardiotoxin injured muscle, phosphorylated CREB was enriched in regenerating areas of *Dmd^mdx^* skeletal muscle from 4-week old animals (Figure 1D). Moreover, *Sik1* mRNA was increased in muscle tissue from 4-week old dystrophin-deficient animals compared with age-matched wild-type control mice (Figure 1E). *Nr4a2* mRNA was also abundant in regenerating regions of skeletal muscle from 28 day-old *Dmd^mdx^* mice (Figure 1F). This pattern was markedly similar to the *Nr4a2* expression pattern we observed in skeletal muscle after cardiotoxin injury. Thus, CREB phosphorylation and transcriptional activity are enriched in regenerating skeletal muscle after acute injury or degeneration due to genetic mutation.

### CREB is activated in both myogenic precursor cells and regenerating myofibers

Many cell types are activated in regenerating skeletal muscle [1], so we performed dual-labeling experiments to identify the cell type in which CREB is activated in injured skeletal muscle. Because cardiotoxin-induced CREB target gene activation coincided with the peak time point of myoblast proliferation, we hypothesized that CREB becomes activated in proliferating myoblasts. The strong unspecific staining with mouse antibodies on necrotic cardiotoxin-injured tissue precluded reliable dual-label analysis in this model (not shown). As expected, we observed enriched phospho-CREB in regenerating areas of *Dmd^mdx^* muscle (4-week old) by immunofluorescence (Figure 1G and Figure S1C). Consistent with our hypothesis, we observed that some, but not all, Pax7-expressing satellite cells in regenerating *mdx* muscle contained high levels of phosphorylated CREB (Figure 1G and Figure S1C, arrowheads). Phosphorylated CREB was not limited to this cell type. Indeed, we observed strong staining in newly regenerated myofibers, which we identified by the characteristic central nuclei (Figure 1G and Figure S1C, dotted circles). It is possible that the unidentified mononucleate cells with high phospho-CREB staining are differentiating myocytes that lost Pax7 expression or are infiltrating macrophages and neutrophils. The strong phospho-CREB staining in newly regenerated myofibers suggests that CREB may play a role in differentiating myocytes during myofiber regeneration. This notion is consistent with previous observations that CREB activity is elevated during myogenic differentiation of C2C12 myoblasts [8]. Further experiments will be required to fully identify the cell type(s) within injured muscle in which CREB and its target genes are activated. Nonetheless, our data show for the first time that CREB phosphorylation is induced in myogenic precursors and nascent myofibers in regenerating skeletal muscle.

### Factors released from crushed muscle activate CREB in primary skeletal myoblasts

**Figure 2:**
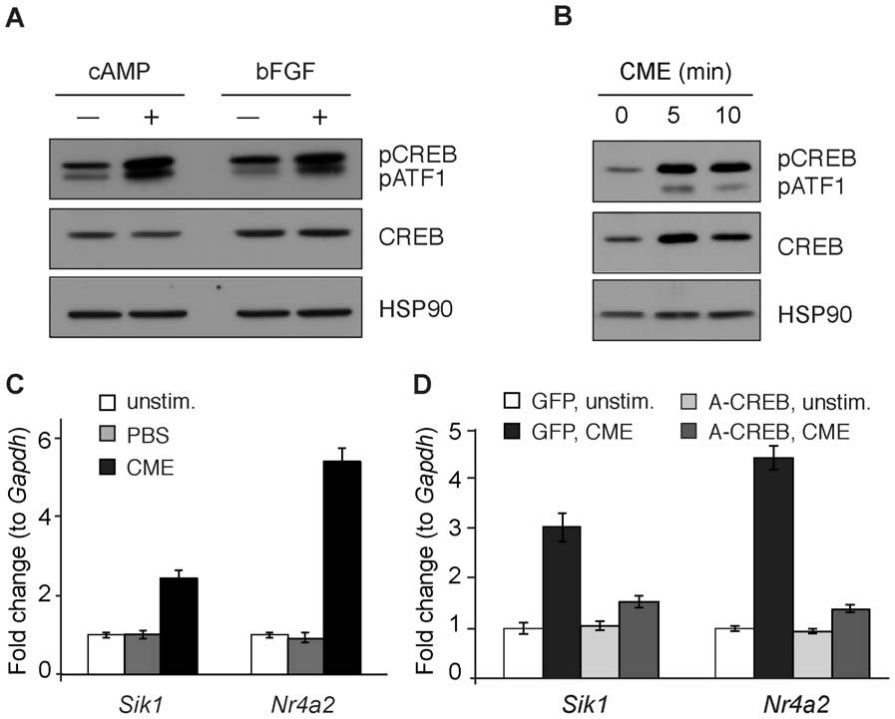
CREB is activated by crushed muscle extract in primary myoblasts. A) Phospho-CREB (pCREBS133), pATF1, total CREB and HSP90 in primary skeletal myoblasts treated with FSK/IBMX (cAMP) or bFGF for 10 min. B) pCREB, total CREB and HSP90 in primary skeletal myoblasts treated with crushed muscle extract (CME) for 0, 5 and 10 min. C) Amounts of *Sik1* and *Nr4a2* mRNA in primary skeletal myoblasts left untreated or incubated 1 h with PBS or CME. D) A-CREB expression in primary skeletal myoblasts blocks *Sik1* and *Nr4a2* induction by CME (1 h). In C and D, mRNA is normalized to *Gapdh*, represented as fold change above unstimulated (± stdev). Representative of 3 experiments. For D, treatments were normalized to GFP, unstimulated for each target gene.

Having observed elevated CREB activity in regenerating skeletal muscle, we hypothesized that after injury, CREB contributes to myoblast activation and/or differentiation. To specifically investigate CREB action in myogenic cell populations, we cultured primary mouse myoblasts, which comprise the cell type that divides and differentiates to form new myofibers. As expected, CREB phosphorylation on Ser133 in these cells was induced by application of forskolin (FSK), which elevates intracellular cAMP, or basic fibroblast growth factor (bFGF), which is a potent myoblast mitogen (Figure 2A). To directly test whether CREB is activated in myoblasts in response to stimuli released from damaged muscle, we treated primary myoblasts with extracts from mouse skeletal muscles crushed *ex vivo*. Similar preparations have previously been shown to induce satellite cell activation [22], proliferation [23] and migration [10]. Crushed muscle extract (CME) rapidly induced CREB phosphorylation in myoblasts (Figure 2B and Figure S2A). Intriguingly, we consistently recovered more total CREB after 5 and 10 minutes of CME treatment compared to untreated control myoblasts (Figure 2B and Figure S2B), although the molecular mechanism is unknown. Acute changes in total CREB levels are not commonly observed upon acute cellular treatments, such as FSK or bFGF (Figure 2A, center). In other cell types, CREB translation [24] or degradation [25], [26] can be modulated by extracellular stimuli on the order of hours. Possible regulation of CREB protein abundance in myocytes will be an interesting avenue for further study.

CREB phosphorylation is necessary but not sufficient for activation of CREB target genes [27]. We therefore tested whether CREB phosphorylation in CME-treated myoblasts correlates with increased CREB transcriptional activity. In agreement with increased CREB(S133) phosphorylation, the CREB target genes *Sik1* and *Nr4a2* increased in primary myoblasts treated for 1 hour with CME (Figure 2C). We used the dominant-negative CREB polypeptide A-CREB [28] to verify that CREB is necessary for *Sik1* and *Nr4a2* mRNA activation by crushed muscle extract. Infection of skeletal myoblasts with adenovirus expressing GFP had little effect on activation of *Sik1* and *Nr4a2* transcription in response to cAMP-inducing agents or crushed muscle extract (Figure 2D and Figure S3). By contrast, adenoviral delivery of A-CREB severely blunted induction of both genes (Figure 2D and Figure S3). These findings are consistent with activation of CREB target genes after cardiotoxin injection *in vivo* and support the notion that at least part of the gene induction we observed in injured areas of skeletal muscle occurred in myoblasts. It is unknown what protein or small molecules in CME activates CREB in myoblasts. Numerous growth factors that could induce CREB phosphorylation are present in CME, including basic fibroblast growth factor (bFGF) [23] and an unidentified G-protein coupled receptor ligand [10]. Our data suggest that one of these signals or an unknown factor released from injured skeletal muscle activates CREB in myoblasts within damaged muscle.

### CREB-YF mice have normal muscle structure

**Figure 3:**
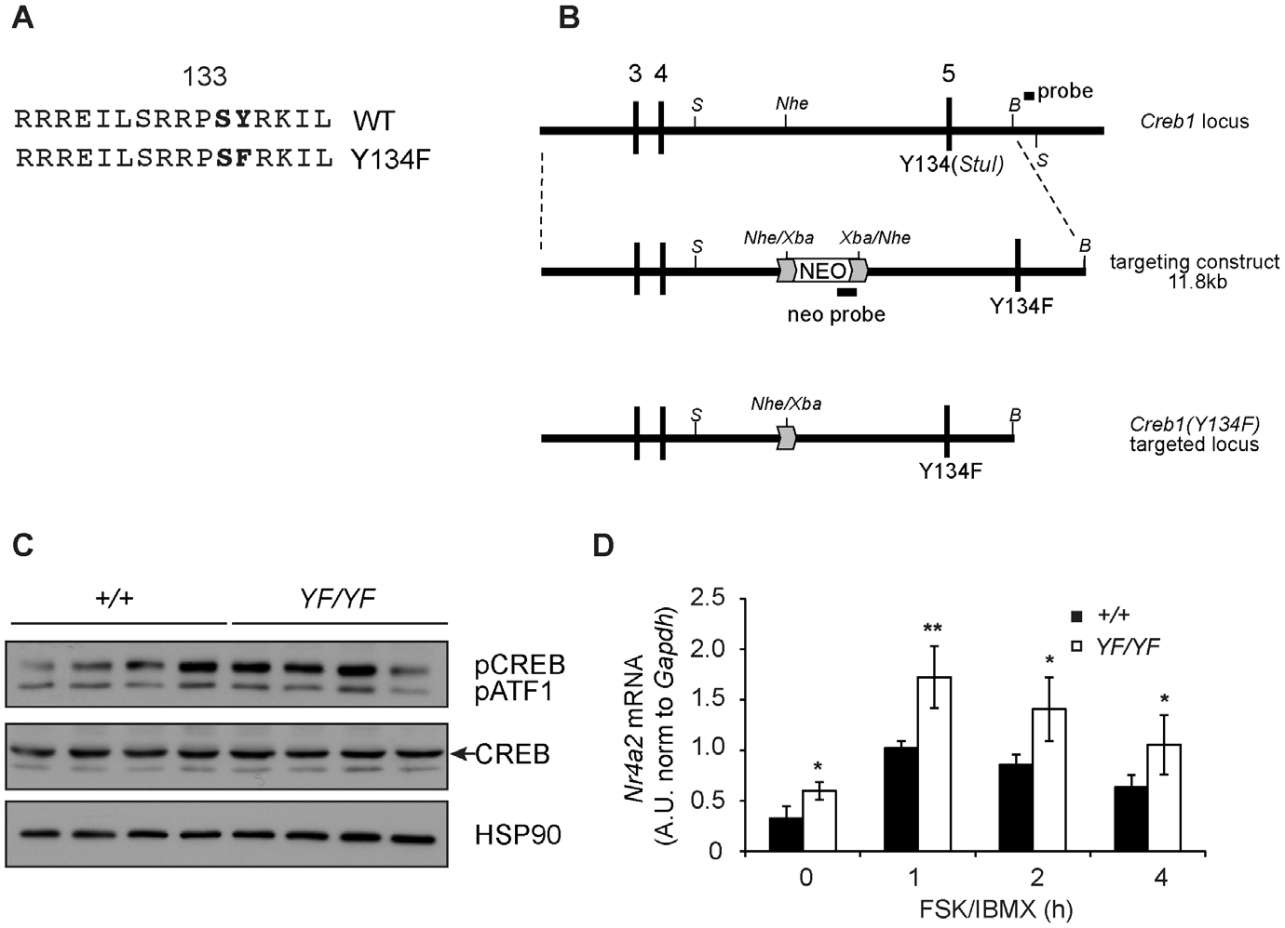
CREB-YF enhances myoblast response to cAMP. A) Amino acid sequence of CREB PKA phosphorylation site (Ser133) showing Y134F mutation. B) Targeting strategy for CREB-YF knock-in strain. Mouse *Creb1* locus with Y134 in exon 5 (top); targeting construct inserts neo cassette in intron 4 and Y134F/*StuI* mutation (middle); targeted locus contains Y134F mutation. S, B: *StuI*, *BamHI* restriction sites. Southern probes indicated. C) Phospho-CREB, CREB and HSP90 protein in gastrocnemius muscle from *Creb^+/+^* and *Creb^YF/YF^* mice. *n = *4 per genotype. D) *Nr4a2* mRNA in primary skeletal myoblasts from wild-type and CREB-YF mice, stimulated with FSK/IBMX for indicated times. *Nr4a2* normalized to *Gapdh*, expressed in arbitrary units (A.U.). **, *p*<.01; *, *p*<.05 between genotypes. Average of 3 experiments ± stdev.

Phosphorylation of CREB on Ser133 promotes recruitment of CBP/p300 and loosening of chromatin at target gene promoters [6]. Mutation of the adjacent Tyr134 to Phe (CREBY134F, Figure 3A) results in enhanced PKA phosphorylation and CBP recruitment [29]. CREB(Y134F) therefore acts as a gain-of-function mutant when over-expressed in cells [9], [30], [31]. To investigate physiologic effects of activated CREB *in vivo* without over-expression, we knocked the Y134F mutation into the *Creb1* locus in mice (Figure 3B and Figure S4). *Creb^+/+^*, *Creb^+/YF^*, and *Creb^YF/YF^* mice were recovered at the expected Mendelian frequency (Table S1). Wild-type and knock-in mice were visually indistinguishable, and CREB-YF homozygotes exhibited no phenotypic abnormalities. We verified that the YF mutation did not alter CREB protein expression in skeletal muscle (Figure 3C). In unchallenged animals, we observed no statistically significant difference in amounts of phospho-CREB between adult *Creb^+/+^* and *Creb^YF/YF^* skeletal muscles (Figure 3C and Figure S5). Histological analysis revealed no differences between skeletal muscles of WT and YF littermates in terms of size or fiber type distribution (not shown). By contrast, expression of CREB-YF in C2C12 myoblasts induces expression of *follistatin*, which promotes myoblast fusion and myotube hypertrophy *in vitro* [9]. Additionally, CREB binds directly to the *cyctochrome c* [32] and *Ppargc1a* (encoding PGC1-alpha) promoters [33], and forced expression of CREB co-activators promotes mitochondrial biogenesis in cultured myocytes [34]. Although we measured a modest increase in *Ppargc1a* mRNA levels in *Creb^YF/YF^* muscle, we observed no difference in myofiber succinate dehydrogenase activity, exercise endurance or voluntary wheel running in these mice (not shown). We conclude that although CREB may be involved in muscle responses to exercise, endogenous expression of CREB-YF does not lead to an increase in CREB activity sufficient to promote muscle hypertrophy or mitochondrial biogenesis *in vivo*.

To assay CREB-YF transcriptional activity, we monitored CREB target gene expression in primary myoblasts from *Creb* ^+/+^ and *Creb^YF/YF^* littermates. Under basal conditions, we observed a statistically significant increase in *Nr4a2* mRNA levels (Figure 3D). When stimulated with saturating concentrations of FSK, primary myoblasts of both genotypes had identical maximal expression of *Nr4a2* mRNA (not shown). However, we observed stronger *Nr4a2* mRNA induction in *Creb^YF/YF^* myoblasts compared to controls upon exposure to a low concentration of FSK (Figure 3D). Thus, CREB(Y134F) is not a constitutively active mutant, but is more sensitive to PKA signaling as previously shown [29]. This is underscored by the fact that *Nr4a2* mRNA levels decline after prolonged FSK treatment in both *Creb* ^+/+^ and *Creb^YF/YF^* myoblasts (Figure 3D).

### Activated CREB promotes myoblast proliferation

**Figure 4:**
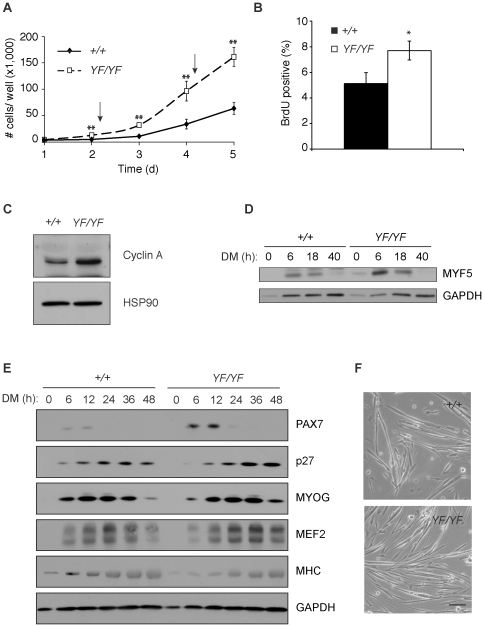
Activated CREB promotes myoblast proliferation and differentiation. A) Growth curve assay of *Creb^+/+^* and *Creb^YF/YF^* myoblasts over 5 days. Arrows, medium added. **, *p*<.01 between genotypes. B) Percent of BrdU-positive nuclei in asynchronous myoblast cultures. Means of 10 fields among 3 replicates. *, *p<*.05. C) CyclinA and HSP90 protein in asynchronous myoblasts. D) Myf5 and GAPDH control in differentiating *Creb^+/+^* and *Creb^YF/YF^* myocytes. E) Muscle-specific proteins in *Creb^+/+^* and *Creb^YF/YF^* myocytes after the indicated times in differentiation medium (DM). MYOG, myogenin; MHC, myosin heavy chain; GAPDH loading control. F) Phase contrast images of 2 day differentiated *Creb^+/+^* and *Creb^YF/YF^* myotubes. Bar, 100 µm.

Because we observed striking induction of CREB activity in regenerating areas of damaged skeletal muscle (Figure 1), we assessed the effects of the CREB-YF gain-of-function mutation on myoblast proliferation and differentiation, two processes that occur as part of muscle regeneration. In growth-curve assays, CREB-YF myoblasts proliferated faster than CREB-WT myoblasts (Figure 4A). Moreover, BrdU-labeling revealed more dividing myoblasts in primary cultures from *Creb^YF/YF^* muscle compared with *Creb^+/+^* control muscle (Figure 4B). This finding is consistent with previous reports showing reduced cell proliferation in somites of *Creb^−/−^* embryos [2]. To explore a potential transcriptional target underlying the enhanced proliferation, we measured expression of CREB target genes implicated in proliferation. We observed an increase in *Ccna2* mRNA in primary myoblasts from *Creb^YF/YF^* mice (not shown), which is also evident at the protein level (Figure 4C). CREB binds to the mouse *Ccna2* promoter [35], suggesting that CREB may directly influence cell cycle progression in myoblasts. Notably, in response to cardiotoxin injection in mouse muscle, *Ccna2* expression and myoblast proliferation are maximal after 3 days [17], coincident with peak expression of the CREB transcriptional targets *Sik1* and *Nr4a2* (Figure 1B).

Paradoxically, CREB has also been implicated in myogenic differentiation through multiple transcriptional targets. During development, a non-canonical Wnt1-cAMP-PKA-CREB pathway drives myogenesis in part via activation of *Myf5* expression [2]. The mouse *Myf5* promoter contains several conserved CREB binding sites and a TATA box [2], [13], so it is likely that CREB directly induces *Myf5* expression. However, CREB binding to the *Myf5* promoter has not been demonstrated. We tested whether CREB-YF is sufficient to drive *Myf5* expression and myogenic differentiation in primary myoblasts. In spite of the enhanced proliferation we observed when CREB-YF myoblasts were cultured in growth medium (Figure 4A, B), CREB-YF cells exhibited stronger upregulation of MYF5 protein within 6 hours of incubation in differentiation medium (Figure 4D and Figure S6A). Similarly, Pax7 was transiently upregulated in both the CREB-WT and CREB-YF cells, but to a greater extent in the CREB-YF cells (Figures 4E and Figure S6B). We do not know if the increased abundance of MYF5 and Pax7 proteins in CREB-YF cultures reflects the presence of more progenitor cells due to proliferation or elevated expression due to enhanced CREB activity on the *Myf5* or *Pax3* promoters [2].

We tested additional cell cycle and myogenic markers in the differentiating CREB-WT and CREB-YF cultures. The cell cycle inhibitor p27 peaked approximately 12 hours later in differentiating CREB-YF myocytes than in CREB-WT controls (Figure 4E and Figure S6C), perhaps reflecting enhanced proliferation in the CREB-YF cultures after plating. Intermediate and late myogenic markers (myogenin, MEF2, myosin heavy chain) increased with similarly delayed kinetics in CREB-YF myotubes compared to controls (Figure 4E and Figure S6D-F). The peak amounts of these proteins were similar between the two genotypes, suggesting that any promotion of myogenesis by CREB occurs early, possibly as a result of *Myf5* induction. *Creb^YF/YF^* myoblasts ultimately differentiated normally into myotubes that were morphologically indistinguishable from *Creb^+/+^* cells (Figure 4F). The slight differences in myotube density in Figure 4F may have resulted from enhanced proliferation of CREB-YF myoblasts in the 18 hours between plating and addition of differentiation medium. Together, our findings show that activated CREB drives both myoblast proliferation and early steps in myogenic differentiation, perhaps through direct regulation of genes that drive the cell cycle and myogenesis, respectively.

### CREB promotes proliferation and muscle regeneration in mice

**Figure 5:**
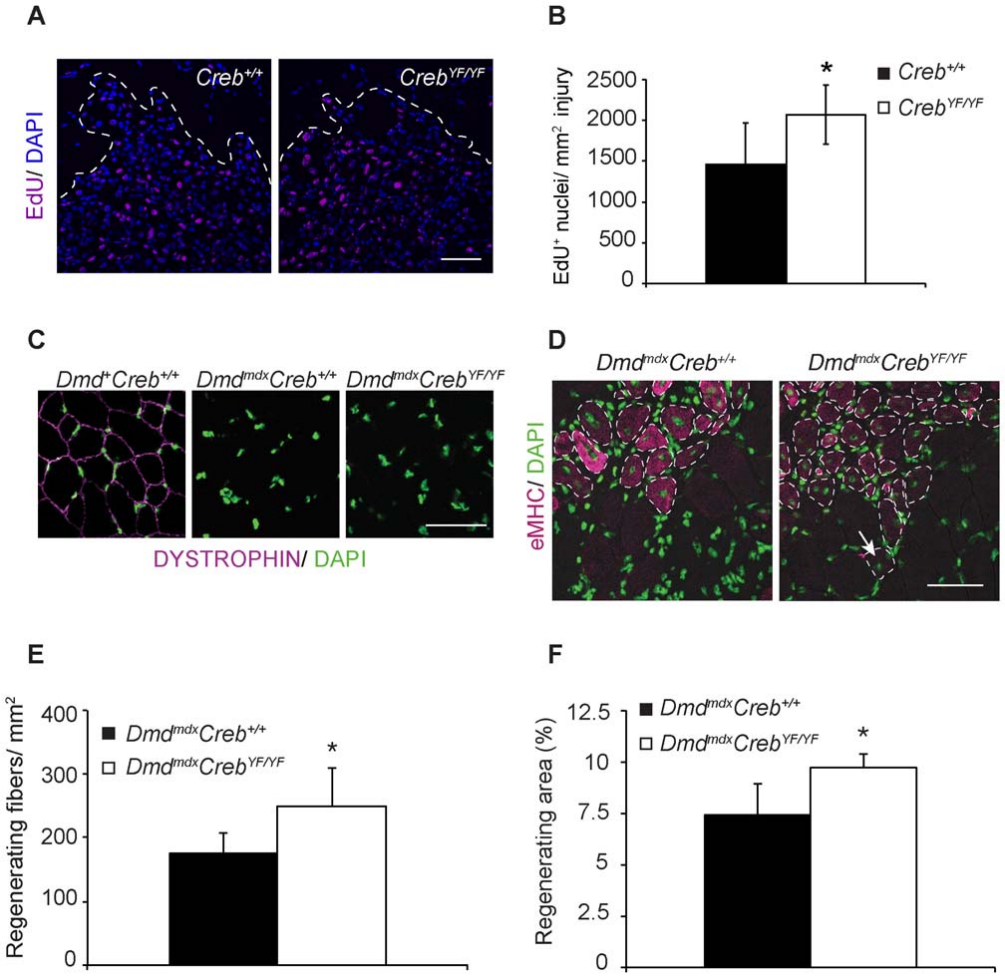
CREB promotes proliferation and muscle regeneration in mice. A) EdU-positive nuclei (magenta) and all nuclei (DAPI, blue) in injured region (below dotted line) of adult male *Creb^+/+^* and *Creb^YF/YF^* mice 5 days after CTX injection with EdU delivery on days 2–4 after cardiotoxin. B) Average number of EdU-positive nuclei per injured area (mm^2^) in 5 fields per mouse. *n = *5 mice per genotype, *, *p*<0.05 by 2-tailed paired *t*-test. C) Anti-dystrophin immunohistochemistry in gastrocnemius sections from 4-week old CREB-YF-*mdx* mice of indicated genotypes. Dystrophin, magenta; DAPI, green. D) Confocal micrographs of regenerating myofibers in cross-sections of CREB-YF-*mdx* gastrocnemius tissue from 4-week old mice visualized by embryonic myosin heavy chain (eMHC, magenta, broken outlines) and central nuclei (DAPI, green, arrow). E) Number of regenerating myofibers per area (±stdev) in fields representing an entire cross-section of the gastrocnemius muscle from animals of the indicated genotypes (4-week old). *n = *6 *Dmd^mdx^Creb^+/^* ^+^, 5 *Dmd^mdx^Creb^YF/YF^*; *, *p*<.05. F) Percent regenerating myofiber area (± stdev) in gastrocnemius tissue. *n* = 3 *Dmd^mdx^Creb^+/^* ^+^, 4 *Dmd^mdx^Creb^YF/YF^*; *, *p*<.05. Bars, 50 µm.

We have shown that CREB phosphorylation and CREB target gene expression are elevated in damaged skeletal muscle and that activated CREB promotes myoblast proliferation and expression of early myogenic markers in primary myocytes. Next, we took advantage of our *in vivo* CREB gain-of-function mouse model to determine how increased CREB activity might affect muscle repair. We first tested whether CREB-YF is sufficient to promote proliferation *in vivo*, as it did in cultured myoblasts. In juvenile animals, we observed no difference in the number of Pax7-positive nuclei between *Creb^+/+^* and *Creb^YF/YF^* animals (not shown). Thus, CREB-YF does not increase the number of resident muscle progenitor cells in the postnatal growth period. To test whether CREB-YF promotes proliferation after acute muscle injury, we injected age-matched adult male CREB-WT and CREB-YF animals with cardiotoxin to induce a synchronous wave of muscle degeneration, myoblast proliferation and regeneration. On days 2, 3 and 4 after injury, we administered EdU to label newly synthesized DNA in proliferating cells [36]. On day 5 after injury, we collected and analyzed the injured tissue for EdU incorporation in the injured areas (Figure 5A). As expected, both *Creb^+/+^* and *Creb^YF/YF^* mice had many proliferating cells in the injured regions, which were evident as large areas of mononucleate cells (Figure 5A, dashed outline). The total injured area in fields of *Creb^+/+^* muscle was similar to that in *Creb^YF/YF^* muscle (Figure S7). However, *Creb^YF/YF^* mice had a significant increase in the number of EdU-positive nuclei per injured area relative to *Creb^+/+^* controls (Figure 5B). This finding is consistent with our *in vitro* data showing elevated proliferation in cultured CREB-YF myoblasts.

We tested effects of the *Creb^YF^* allele on muscle regeneration by crossing the *Creb^YF/YF^* mice with *mdx* mice. The CREB-YF allele did not restore dystrophin expression (Figure 5C), and degeneration proceeded normally in CREB-YF-*mdx* mice (plasma creatine kinase: *Dmd^mdx^Creb^+/+^* 1,961±950 U/L; *Dmd^mdx^Creb^YF/YF^* 2,295±267; *p = *.52). We visualized regenerating myofibers in muscle sections by embryonic myosin heavy chain (eMHC) staining and centrally located nuclei (Figure 5D) and observed a significant increase in the number of regenerating myofibers from CREB-YF-*mdx* compared to CREB-WT-*mdx* controls (Figure 5E). Moreover, the total cross-sectional area of actively regenerating myofibers was ∼30% greater in *Dmd^mdx^Creb^YF/YF^* than control *Dmd^mdx^Creb^+/+^* mice (Figure 5F). These data demonstrate that genetic activation of CREB promotes myofiber regeneration in dystrophic *mdx* mice. It is possible that the enhanced regeneration we observe results from enhanced proliferation at an earlier time point. Indeed, we noted a significant increase in the proportion of small diameter myofibers in some CREB-YF-*mdx* mice compared to CREB-WT-*mdx* controls, but this trend varied among individual animals (not shown). Alternatively, the skew to smaller myofibers could result from a defect in myotube fusion, although we did not observe overt fusion defects in differentiating myocyte cultures. It is also noteworthy that the intensity of myosin heavy chain staining varied within regenerating regions, as shown in Figure 5D. Replacement of the embryonic myosin heavy chain isoform by adult isoforms at later stages of myofiber regeneration could account for the reduced overall intensity [37] if CREB-YF-*mdx* myofibers were regenerating faster than CREB-WT-*mdx* myofibers. Variance in eMHC intensity did not impact our quantification, as we included all eMHC-positive and centrally nucleated myofibers (even those with no eMHC staining).

Because our genetic strategy results in CREB-YF expression in all cells, enhanced CREB activity in other cell types may contribute to the regenerative phenotype. This is particularly true of immune cells in which CREB has multiple roles including activation of cytokine gene expression and promotion of survival, migration and proliferation [38]. We do not know at which stage in myogenesis CREB acts to promote regeneration. Many nuclei in regenerating *mdx* muscle have readily detectable phospho-CREB. Some of those nuclei express the satellite cell marker Pax7, but many other nuclei do not (Figure 1G and Figure S1C). The strong phospho-CREB staining in central nuclei of newly regenerated myofibers suggests that perhaps CREB is activated at multiple stages of muscle regeneration. Further studies including lineage-tracing analysis will be necessary to resolve this question.

Our results show that genetic activation of CREB is sufficient to induce both myoblast proliferation and differentiation *in vitro* and proliferation and muscle regeneration *in vivo*. It is not known which CREB target genes mediate these phenotypes. The distinct cellular phenotypes we observed in cultured primary myoblasts correlate with differential expression of the cell cycle regulator *Ccna2* as well as accumulation of the early myogenic marker MYF5, which have been previously implicated as CREB target genes [2], [35]. These data suggest that multiple CREB target genes may be involved in the complex process of muscle regeneration. Additional genetic studies will reveal the molecular mechanisms and target genes underlying the effects of CREB on proliferation and regeneration in injured skeletal muscle.

Together our findings support a model in which CREB is activated in myoblasts and regenerating myofibers by stimuli released within damaged skeletal muscle tissue, whereupon CREB promotes myoblast proliferation and contributes to myofiber regeneration. We posit that the mechanisms by which CREB participates in skeletal muscle regeneration are distinct from those in axon regeneration [39] because muscle regeneration involves activation, proliferation and differentiation of resident quiescent stem cells. We are just beginning to uncover CREB-dependent molecular mechanisms that may contribute to the salutary effects of cAMP signaling on muscle strength [5] and possibly regeneraftion in humans and mice with muscular disease.

## Methods

### Primary skeletal myoblasts

Myofiber-associated satellite cells and myoblasts were prepared from neonatal mouse limb muscles as described [40]. Myoblasts were seeded on Matrigel in DMEM-F10, 20% FCS, 2.5 ng/mL bFGF (Peprotech). Cells were differentiated in DMEM, 2% horse serum.

### Cell treatments

Myoblasts were treated with forskolin (FSK, EMD Chemicals)/3-isobutyl-1-methylxanthine (phosphodiesterase inhibitor, IBMX, Sigma) in DMSO at final concentrations of 1 µM FSK/1.8 µM IBMX. For bFGF stimulation, myoblasts were starved for 3 h in DMEM-F10, 3% FBS then treated with recombinant bFGF (2.5 ng/mL). For crushed muscle extract (CME), isolated gastrocnemius muscles of adult mice were crushed with blunt forceps and incubated in PBS with tumbling 1.5 h, 4°C. Myoblasts were treated with CME (250 µg/mL) or PBS (equal volume). Myoblasts were labeled with BrdU (10 µM, 1 h) and analyzed by immunofluorescence, counting >800 cells per condition among 3 sets of myoblasts.

### Adenovirus

Recombinant adenovirus encoding GFP or Flag-A-CREB was propagated in HEK-293 cells and purified by ultracentrifugation on CsCl_2_ density gradients and dialyzed extensively into PBS/10% glycerol as described [41]. Freshly explanted myoblasts were allowed to adhere overnight and then infected for 6 hours. 24 hours after infection, efficiency was determined by GFP expression. Cells were then treated for 1 hr with FSK/IBMX or CME. The University of Texas Health Institutional Biosafety Committee approved the use of adenovirus (IBC-09-009).

### Protein extracts

Whole cell extracts were prepared from cells and tissue in RIPA buffer (20 mM HEPES pH7.5, 137 mM NaCl, 0.5% TX-100, 0.1% SDS, 0.5% Na-DOC, 0.5 mM EDTA, 5 mM Na_4_P_2_O_7_, 20 mM beta-glycerol phosphate, 50 mM NaF, 1 mM Na_3_VO_4_, PI cocktail) and concentration measured by BCA assay as described [7]. For phospho-CREB and total CREB western blots, samples were resolved on duplicate gels in parallel. pCREB and total CREB were probed on different blots to prevent misinterpretation of data due to residual signal or loss of protein after stripping. HSP90 loading controls were performed for each blot to verify equal loading; shown for the pCREB blots.

### Gene expression

cDNA was prepared and analyzed from total RNA by QPCR as described [7]. Gene-specific RT was used for 18 S rRNA; oligo-dT RT was used for other transcripts. Relative mRNA amounts were normalized in each sample to an internal control and expressed in arbitrary units or fold change. See Table S2 for primer sequences.

### Antibodies

Rabbit monoclonal phospho-CREB(S133) (87G3), CREB (48H2) and p27 (D69C12), Cell Signaling; mouse monoclonal adult myosin heavy chain (MHC) MF20 [42], embryonic (e)MHC F1.652 [43], myogenin F5D [44] and dystrophin MANDRA clone 7A10 [45], Developmental Studies Hybridoma Bank; MEF2 C-21 [46] and HSP90 C-20 [47], Santa Cruz; BrdU clone B44 [48], BD Biosciences; CyclinA2 AF5999, R&D Systems; DyLight549-AffiniPure F(ab')_2_ Donkey anti-mouse IgG (715-506-150) and FITC-AffiniPure F(ab')_2_ Donkey anti-rabbit IgG (711-096-152), Jackson Immunoresearch.

### CREB(Y134F) mice

A targeting vector was constructed from a 10.5-kb genomic fragment encompassing exons 3–5 of mouse *Creb1*. Site-directed mutagenesis was used to introduce the Y134F mutation. *Creb^+/YF^* mice were backcrossed for 10 generations to C57Bl/6 to obtain *Creb* ^+/+^ and *Creb^YF/YF^* mice. See Materials and Methods S1 for details.

### Animals

Animal experiments performed in this study were specifically approved by the IACUC Committees of the Salk Institute (01-047, 04-042) and the University of Texas Health (HSC-AWC-08-125, HSC-AWC-11-096). Breeding of transgenic rodents was also approved by the UT Health Institutional Biosafety Committee (IBC-09-009). C57Bl/6 were purchased from Harlan; ICR from Taconic; *Dmd^mdx^* from Jackson. Cardiotoxin (10 µM, 25 µL per mouse, Sigma) was injected into gastrocnemius muscles of 8–10 wk mice. *Creb^YF/YF^* males and *Dmd^mdx/mdx^* females were crossed to generate the CREB-YF-*mdx* strain. Double heterozygous offspring were intercrossed to obtain homozygous mice. Plasma creatine kinase (CK) activity was measured from EDTA-treated tail blood of 3.5-week old mice with an enzymatic assay (Catachem, Inc) prior to isolation of muscles from the animals of desired genotype (28 days old). Genotyping protocols are provided in Materials and Methods S1. CME was obtained from skeletal muscles of adult ICR mice (8–24 weeks).

### *In vivo* EdU labeling

Male *Creb^+/+^* and *Creb^YF/YF^* mice (8 wk) were anesthetized with isoflurane and gastrocnemius muscles of the right hind limb were treated with topical lidocaine (0.5%) and injected with cardiotoxin (10 µM, 25 µL per mouse, Sigma) on day zero of the experiment. EdU (5 mg/kg in 0.9% saline, Invitrogen) was administered via subcutaneous injection on days 2, 3, and 4 after cardiotoxin injury, and tissue was collected on day 5 of the experiment [36]. Ketoprofen (Butler Animal Health Supply) was administered via subcutaneous injection (5 mg/kg, days 0–1; 2 mg/kg days 2–4). Isolated muscles were flash frozen in OCT in liquid nitrogen-cooled isopentane. 8-µm fresh frozen sections through the muscle belly were collected, fixed for 10 min in 10% buffered formalin and permeabilized in 0.3% TX-100-PBS for 20 min. EdU-labeled DNA was detected by manufacturer's directions using the Click-It EdU Alexa-647 detection kit (Invitrogen). Sections were washed in 1× PBS and counterstained with DAPI (2 µg/mL). Confocal images were collected on a Nikon A1R microscope and all visible EdU-positive nuclei in five fields for each of five animals per genotype were counted using Nikon NIS Elements software. Injured areas were identified by visualization of high concentrations of mononucleate cells in the DAPI channel and measured using Nikon NIS elements software. For each field, the number of EdU-positive nuclei was normalized to the injured area, then averaged by genotype (±stdev). The average injured area in mm^2^ for each genotype was also calculated.

### Immunohistochemistry

Phosphorylated CREB was visualized in 8-µm frozen muscle sections prepared as described for EdU detection. After permeabilization and washing, sections were stained with anti-phospho-CREB antibody (1∶100) using the MOM and ABC-HRP detection kits with NovaRed substrate (Vector Laboratories). Brightfield images were obtained on a Nikon 80i microscope with a color camera and Nikon NIS Elements software using matched light and exposure settings. For fluorescence detection, the blocking and antibody dilution buffer was PBS/10% normal goat serum/0.3% TX-100. Primary antibodies to phospho-CREB and Pax7 (1∶100) were added for 1 hour. Secondary antibody incubations were sequential: 45 min anti-rabbit (1∶500) and 10 min anti-mouse (1∶2000 with 0.05% Tween-20). Sections were washed and counterstained with DAPI (2 µg/mL) prior to imaging on a Nikon A1R confocal microscope with sequential laser scanning.

### *In situ* hybridization

*In situ* hybridization on skeletal muscle sections was performed using an automated system as described [49] with 4 µg/mL Proteinase K. An antisense digoxygenin-labeled riboprobe complementary to the mouse *Nr4a2* transcript (nucleotides 337–934) was synthesized by *in vitro* transcription, used at 300 ng/mL. Negative controls included no probe and sense probe. The sense probe exhibited strong unspecific staining in all tissues examined (embryos and muscle) and was disregarded as an inappropriate control. The *Nr4a2* antisense probe recognized the reported *Nr4a2* expression pattern in E14.5 mouse embryos [18], [50]. Sections were imaged using a Nikon 80i microscope with color camera and Nikon NIS Elements software.

### Muscle regeneration

Cross-sections of fresh frozen tissue were stained with an eMHC antibody using the MOM detection kit (Jackson). Micrographs of tiled fields representing an entire section through the gastrocnemius muscle belly of each mouse were collected (Nikon A1 confocal). Section area, number of regenerating fibers (eMHC positive or central nucleus) and cross-sectional area of individual regenerating fibers were measured with Adobe Photoshop. Areas of myofibers at oblique angles were omitted. The percent regenerating area was the sum of individual myofiber areas divided by the total section area. Values were averaged for age-matched double homozygous animals irrespective of sex.

### Statistics

Significance between means of groups was determined by the 2-tailed Student's *t*-test (Excel).

## Supporting Information

Figure S1 **Localization of phospho-CREB in muscle tissue.** A) Full panel of no primary control (npc) inset shown in Figure 1A. B) Phospho-CREB(S133) staining in contralateral legs of 2 wild-type mice (uninjured and 3d after CTX injury). Bars, 50 µm. C) Phospho-CREB (green) and Pax7 (red) staining in 2 *Dmd^mdx^* mice. Filled arrowheads, Pax7-pCREB double positive nuclei; open arrowheads, Pax7-positive nuclei with low pCREB staining; outlines, regenerating myofibers. Bottom row, no primary control (npc) in the same experiment with matched imaging settings on serial sections of muscle from animal #3. Bars, 10 µm.
(TIF)

Figure S2 **Quantification of western blots in** **Figure 2B** **.** A) Ratio of pCREB/total CREB. B) Ratio of total CREB/HSP90. Data represent averages of normalized intensity among panel shown in Figure 2B and replicate samples. *, *p*<.05; ** *p*<.01. Data are representative of qualitative analysis of 4 independent sets of treated cells.
(TIF)

Figure S3 **A-CREB blunts expression of cAMP-induced genes in primary myoblasts.** CREB target gene mRNA in Ad-GFP or Ad-ACREB infected primary myoblasts treated 1 h with FSK/IBMX (cAMP). mRNA amounts normalized to *Gapdh* internal control, expressed as fold difference to GFP, unstimulated control for each target gene. Data are average ±stdev (measurement error). Data represent 3 independent experiments.
(TIF)

Figure S4 **Southern blot of targeted mouse** ***Creb*** **locus.** Southern blot of *Creb^+/+^* and *Creb^+/YF^* ES cell DNA digested with *BstXI* and hybridized to a 3′ probe external to the targeting cassette. 8.7-kb (*Creb^YF^*) and 7.4-kb (*Creb^+^*) bands indicated.
(TIF)

Figure S5 **Quantification of western blot data shown in** **Figure 3C** **.** Average pCREB/total CREB ratio (±stdev). *n* = 4 mice per genotype.
(TIF)

Figure S6 **Quantification of western blot data shown in** **Figure 4D, 4E** **.** Densitometric ratios for indicated protein/GAPDH control on same blot shown in arbitrary units (A.U.). *Creb^+/+^* (black bars) and *Creb^YF/YF^* (open bars) incubated in differentiation medium (DM) for the indicated times in hours. A) MYF5, B) PAX7, C) p27, D) Myogenin (MYOG), E) MEF2, F) Myosin heavy chain (MHC).
(TIF)

Figure S7 **Average injured area after cardiotoxin treatment.** Average mononucleated area (mm^2^) in cardiotoxin injured muscle 5 days after injury. Average of 5 fields per mouse ±stdev. *n* = 5 mice per genotype.
(TIF)

Table S1 **Summary of offspring statistics from** ***Creb^+/YF^*** **intercrosses.** Sex and genotype data from 20 litters (134 pups) of *Creb^+/YF^* heterozygous intercrosses. Two pups of unknown genotype (but known sex) were excluded from genotype summaries. Expected offspring ratios are shown. Chi squared probability tests were applied with 1 (sex) or 2 (genotype) degrees of freedom with continuity correction as appropriate to determine *p* values.
(PDF)

Table S2 **Oligonucleotide primers utilized for QPCR assays.**
(PDF)

Materials and Methods S1 (PDF)
