# Supplementary figures and images for: CREB Is Activated by Muscle Injury and Promotes Muscle Regeneration
Source: PLoS One. 2011 Sep 13;6(9):e24714. doi: 10.1371/journal.pone.0024714 (PMC3172299; doi:10.1371/journal.pone.0024714)

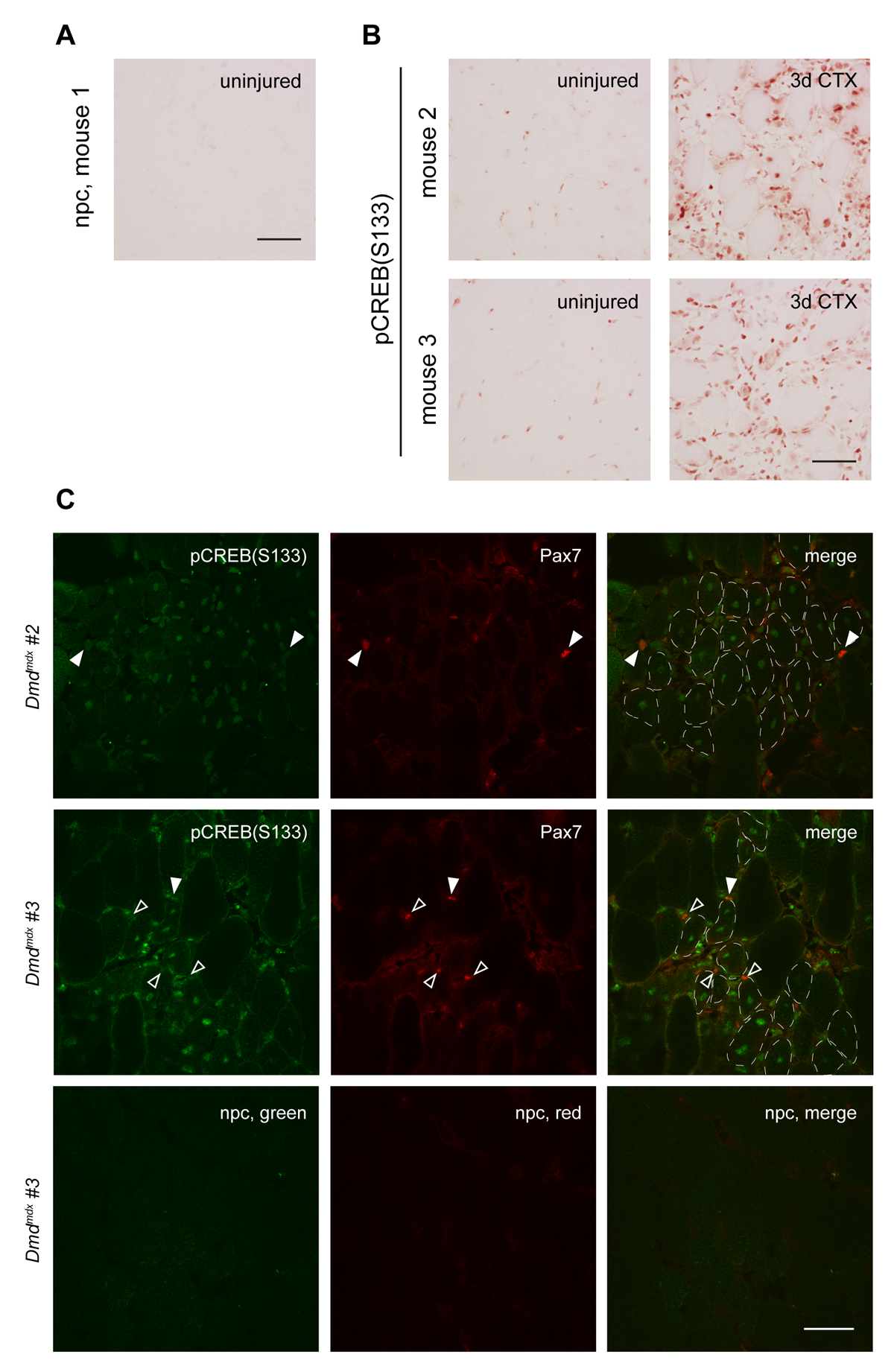

Supplement: Figure S1 — Localization of phospho-CREB in muscle tissue. A) Full panel of no primary control (npc) inset shown in Figure 1A. B) Phospho-CREB(S133) staining in contralateral legs of 2 wild-type mice (uninjured and 3d after CTX injury). Bars, 50 µm. C) Phospho-CREB (green) and Pax7 (red) staining in 2 Dmdmdx mice. Filled arrowheads, Pax7-pCREB double positive nuclei; open arrowheads, Pax7-positive nuclei with low pCREB staining; outlines, regenerating myofibers. Bottom row, no primary control (npc) in the same experiment with matched imaging settings on serial sections of muscle from animal #3. Bars, 10 µm. (TIF) [file pone.0024714.s001.tif]

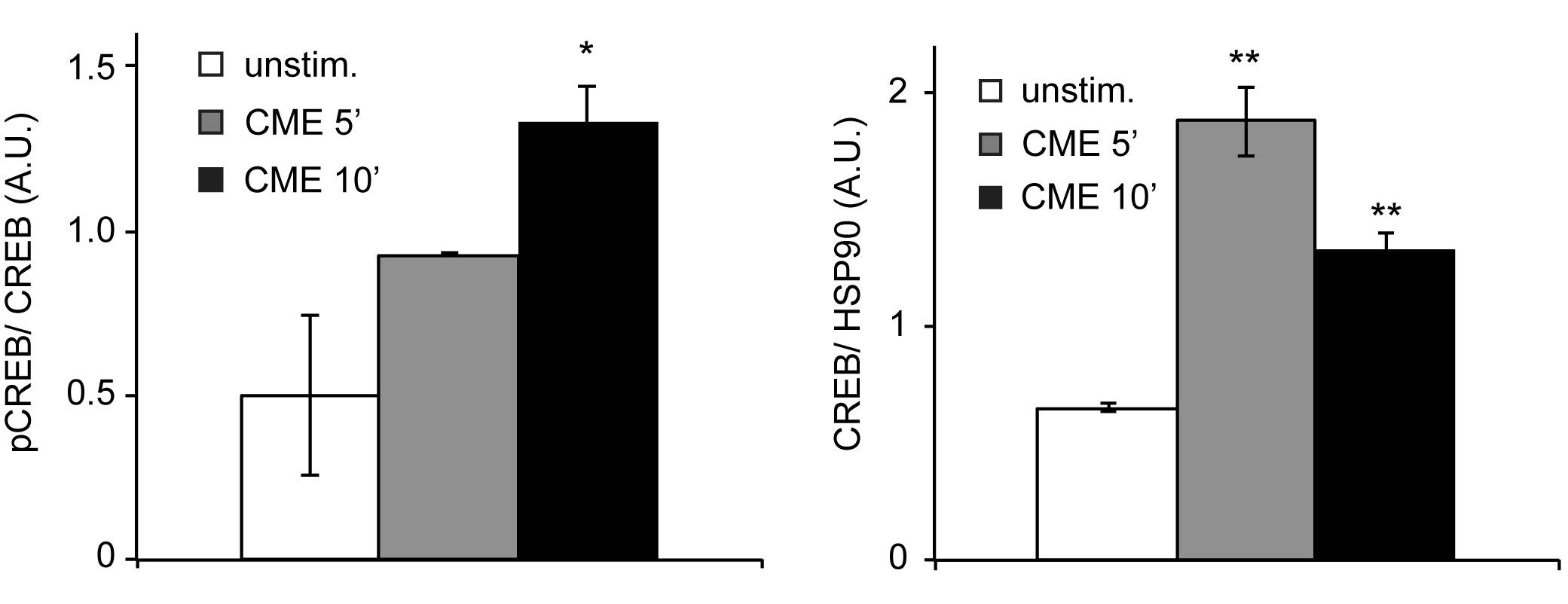

Supplement: Figure S2 — Quantification of western blots in Figure 2B . A) Ratio of pCREB/total CREB. B) Ratio of total CREB/HSP90. Data represent averages of normalized intensity among panel shown in Figure 2B and replicate samples. *, p<.05; ** p<.01. Data are representative of qualitative analysis of 4 independent sets of treated cells. (TIF) [file pone.0024714.s002.tif]

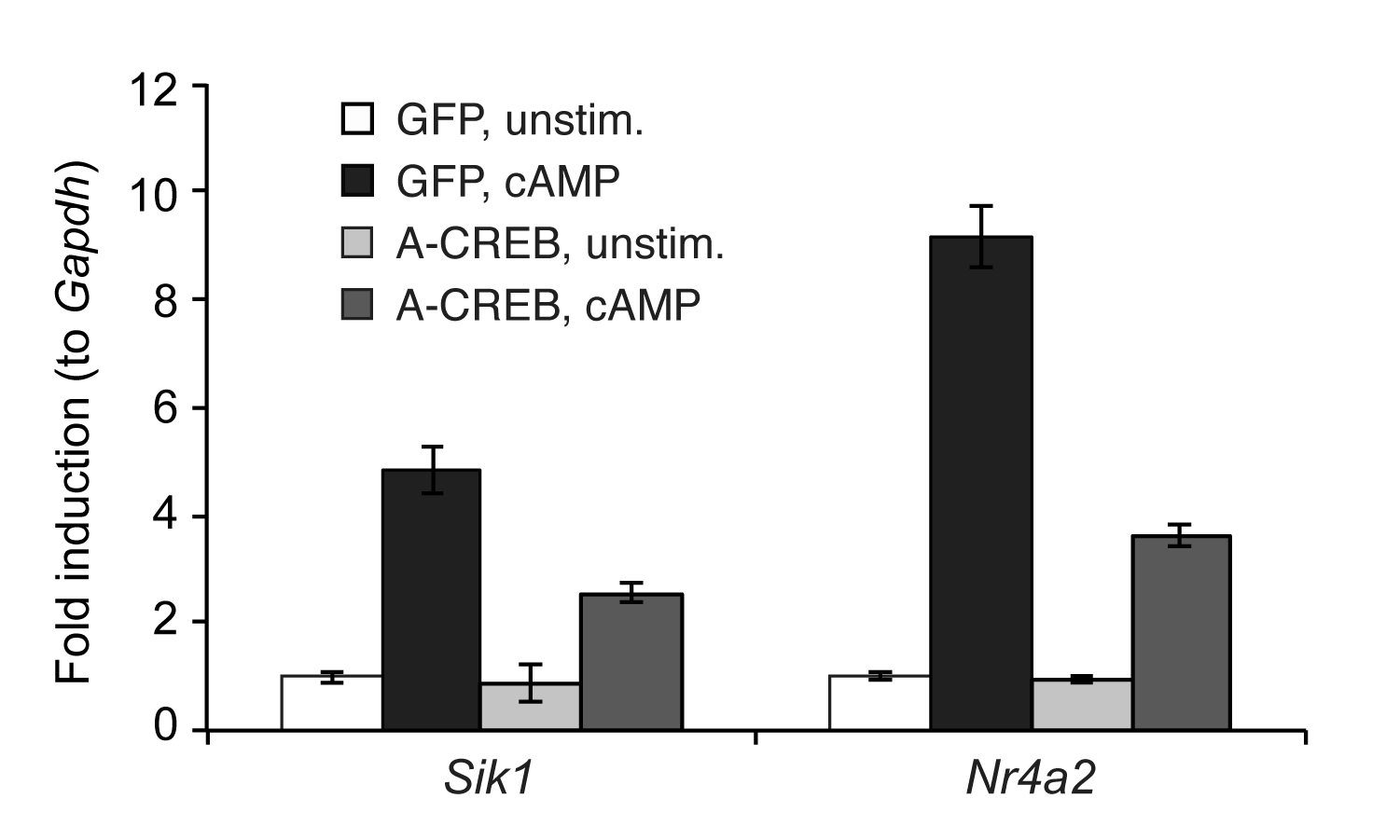

Supplement: Figure S3 — A-CREB blunts expression of cAMP-induced genes in primary myoblasts. CREB target gene mRNA in Ad-GFP or Ad-ACREB infected primary myoblasts treated 1 h with FSK/IBMX (cAMP). mRNA amounts normalized to Gapdh internal control, expressed as fold difference to GFP, unstimulated control for each target gene. Data are average ±stdev (measurement error). Data represent 3 independent experiments. (TIF) [file pone.0024714.s003.tif]

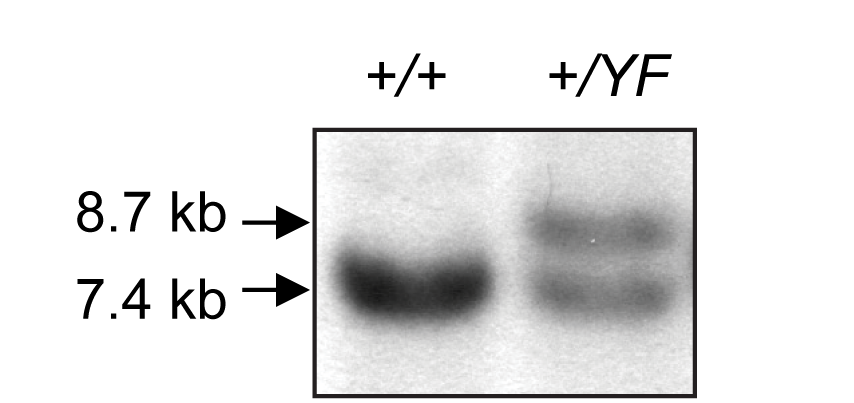

Supplement: Figure S4 — Southern blot of targeted mouse Creb locus. Southern blot of Creb+/+ and Creb+/YF ES cell DNA digested with BstXI and hybridized to a 3′ probe external to the targeting cassette. 8.7-kb (CrebYF) and 7.4-kb (Creb+) bands indicated. (TIF) [file pone.0024714.s004.tif]

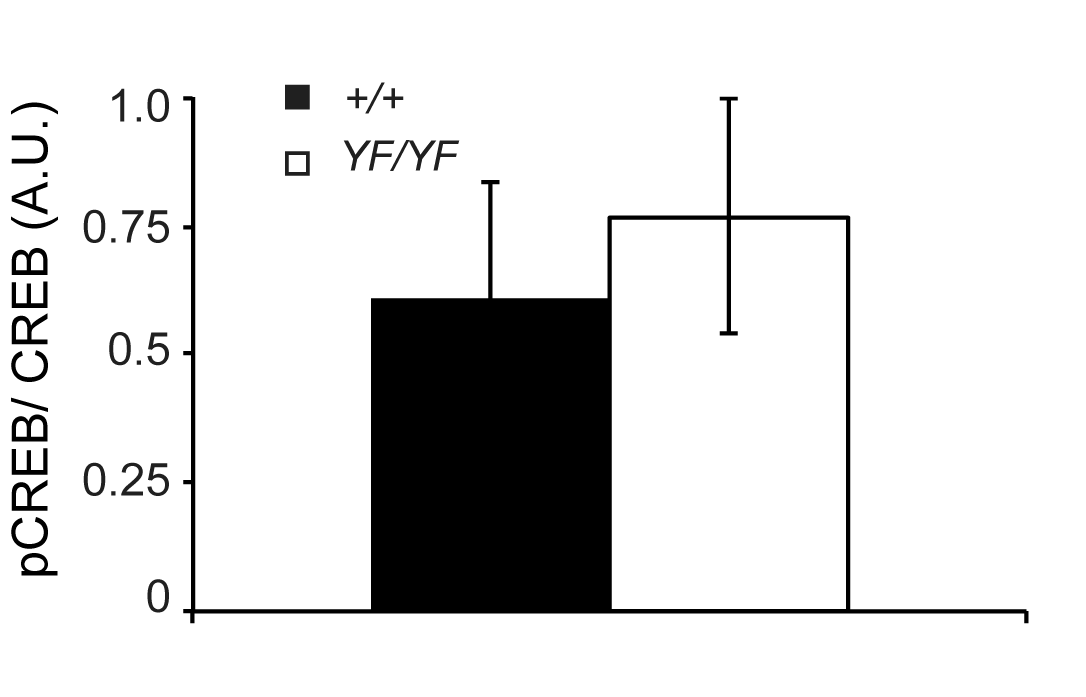

Supplement: Figure S5 — Quantification of western blot data shown in Figure 3C . Average pCREB/total CREB ratio (±stdev). n = 4 mice per genotype. (TIF) [file pone.0024714.s005.tif]

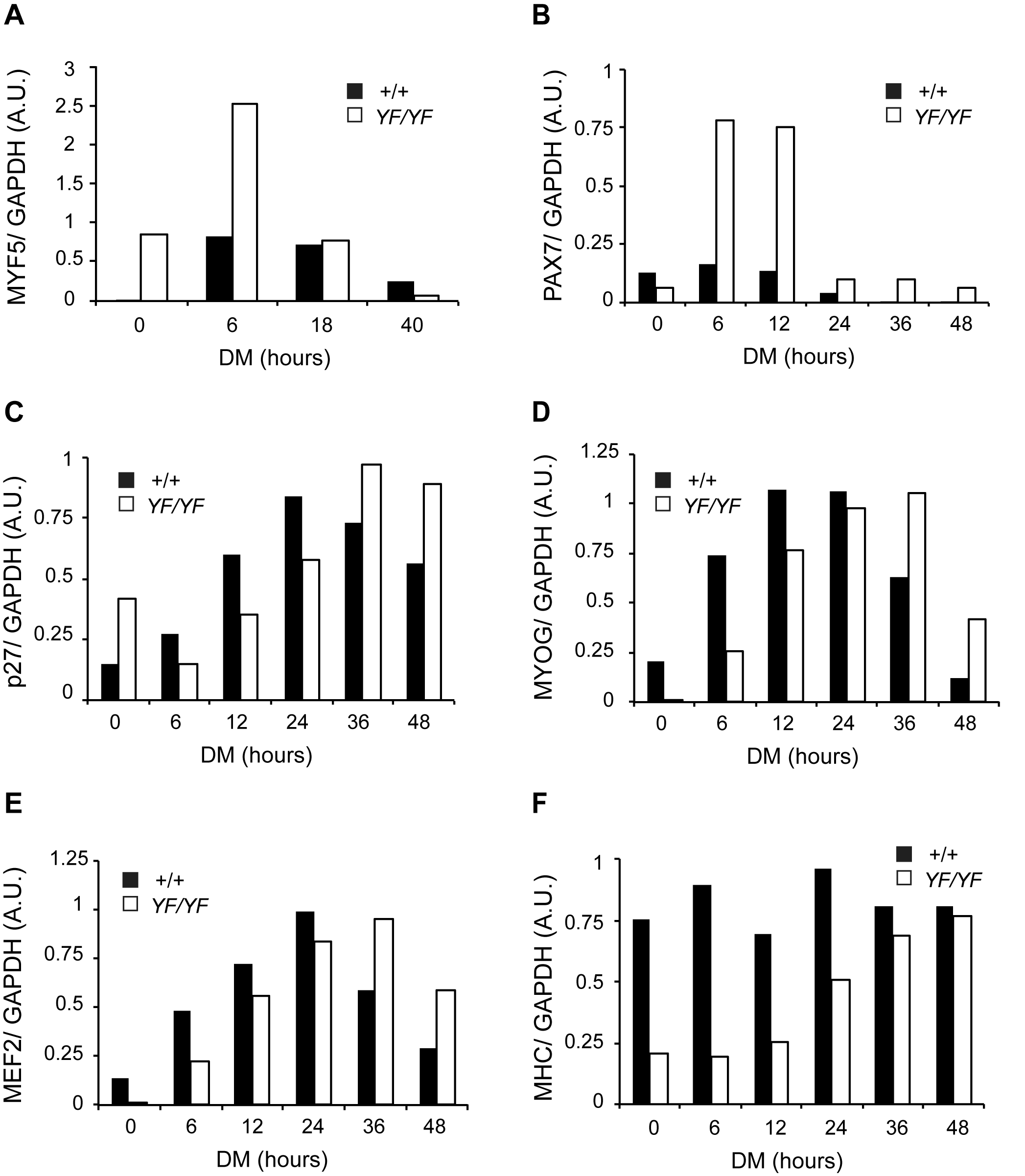

Supplement: Figure S6 — Quantification of western blot data shown in Figure 4D, 4E . Densitometric ratios for indicated protein/GAPDH control on same blot shown in arbitrary units (A.U.). Creb+/+ (black bars) and CrebYF/YF (open bars) incubated in differentiation medium (DM) for the indicated times in hours. A) MYF5, B) PAX7, C) p27, D) Myogenin (MYOG), E) MEF2, F) Myosin heavy chain (MHC). (TIF) [file pone.0024714.s006.tif]

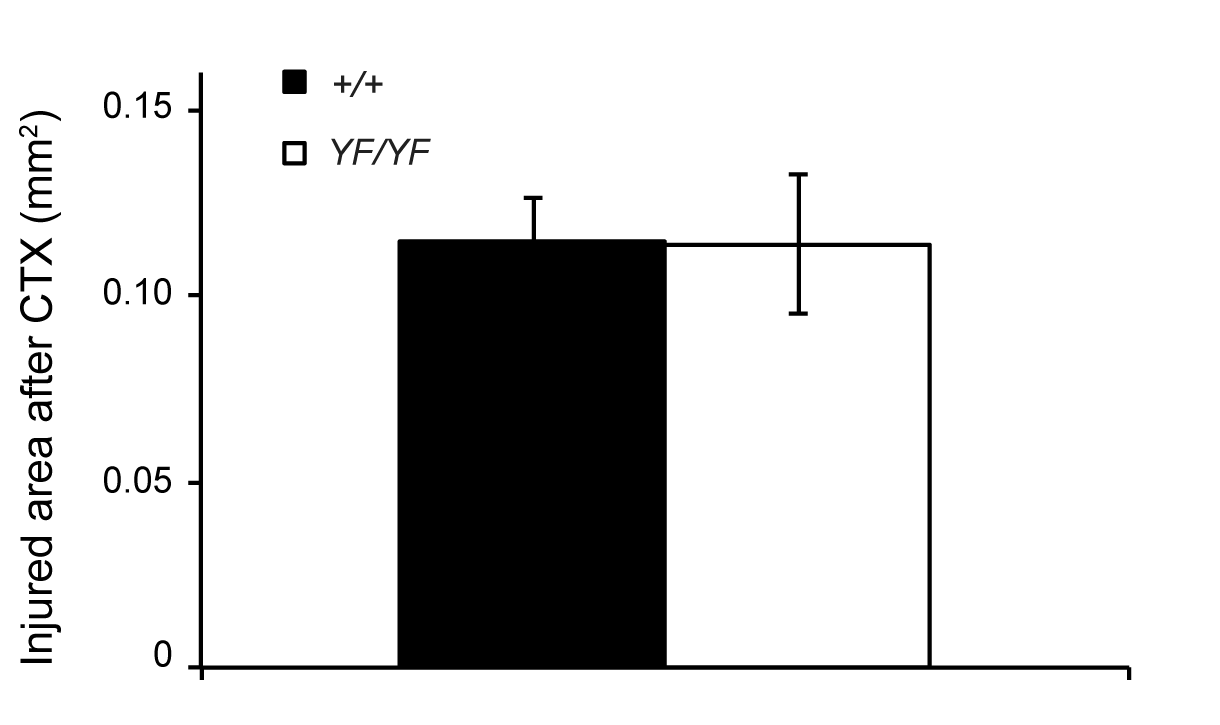

Supplement: Figure S7 — Average injured area after cardiotoxin treatment. Average mononucleated area (mm2) in cardiotoxin injured muscle 5 days after injury. Average of 5 fields per mouse ±stdev. n = 5 mice per genotype. (TIF) [file pone.0024714.s007.tif]
