# Supplementary material for: CREB Is Activated by Muscle Injury and Promotes Muscle Regeneration
Source: PLoS One. 2011 Sep 13;6(9):e24714. doi: 10.1371/journal.pone.0024714 (PMC3172299; doi:10.1371/journal.pone.0024714)
Supplement: Table S1 — Summary of offspring statistics from Creb+/YF intercrosses. Sex and genotype data from 20 litters (134 pups) of Creb+/YF heterozygous intercrosses. Two pups of unknown genotype (but known sex) were excluded from genotype summaries. Expected offspring ratios are shown. Chi squared probability tests were applied with 1 (sex) or 2 (genotype) degrees of freedom with continuity correction as appropriate to determine p values. (PDF) [file pone.0024714.s008.pdf]

	Sex		Genotype		
	**Male**	**Female**	**+/+**	***+/YF***	***YF/YF***
**Offspring distribution (#)**	71	62	38	55	42
**Expected (#)**	67	67	33	66	33
**Offspring distribution (%)**	53%	47%	28%	41%	32%
**Expected (%)**	50%	50%	25%	50%	25%
**Significance (p)**	0.8<p<0.9		p>0.8		

Table S1. Sex and genotype data from 20 litters (134 pups) of $Creb^{+/YF}$ heterozygous intercrosses. Two pups of unknown genotype (but known sex) were excluded from genotype summaries. Expected offspring ratios are shown. Chi squared probability tests were applied with 1 (sex) or 2 (genotype) degrees of freedom with continuity correction as appropriate to determine *p* values.
