# Supplementary material for: CREB Is Activated by Muscle Injury and Promotes Muscle Regeneration
Source: PLoS One. 2011 Sep 13;6(9):e24714. doi: 10.1371/journal.pone.0024714 (PMC3172299; doi:10.1371/journal.pone.0024714)
Supplement: Table S2 — Oligonucleotide primers utilized for QPCR assays. (PDF) [file pone.0024714.s009.pdf]

	Forward (5'-3')	Reverse (5'-3')
Nr4a2	CGCCGAAATCGTTGTCAGTA	CGACCTCTCCGGCCTTTTA
Sik1	ATTGTCCCCATGTTTGTGGT	TACTGCTGCGGTGAGATTTG
Gapdh	AGGTCGGTGTGAACGGATTTG	TGTAGACCATGTAGTTGAGGTCA
**18S rRNA**	GTAACCCGTTGAACCCCATT	GGCCTCACTAAACCATCCAA
**18S rRNA RT primer**		GTTCGACCGTCTTCTCAGC

Table S2. Oligonucleotide primers utilized for QPCR assays.
