# Supplementary material for: CREB Is Activated by Muscle Injury and Promotes Muscle Regeneration
Source: PLoS One. 2011 Sep 13;6(9):e24714. doi: 10.1371/journal.pone.0024714 (PMC3172299; doi:10.1371/journal.pone.0024714)
Supplement: Materials and Methods S1 — (PDF) [file pone.0024714.s010.pdf]

Supplementary Materials and Methods for Stewart, *et al*

Creation of CREB(Y134F) knock-in mice. The *Creb* genomic locus was isolated from a FIXII λ phage library containing ~10-kb fragments of mouse 129Sv genomic DNA (donated by Tony Hunter) by three rounds of screening with standard methods [1] using a radiolabelled 200-bp *EcoRI-XbaI* fragment of pcDNA3-HA-KID corresponding to the CREB kinase inducible domain (exons 5-6) [2]. Positive phage clones were subcloned into the *NotI* site of pBluescript (pBK). One phage clone harboring a 17.5-kb genomic region was truncated to 10.5-kb and 7-kb fragments using *NotI* and *BamHI* restriction sites (pBK-10 and pBK-7) and further manipulated to target the CREB gene in ES cells. pBK-7 retained the genomic DNA 3' to the targeted region. An *XbaI* fragment of pOG277 [3] containing a 1.2-kb loxP-flanked neomycin resistance cassette was cloned into pBK-10 using complimentary ends of an *NheI* restriction site 3.5-kb upstream of *Creb* exon 5. To insert the Y134F mutation while avoiding unspecific point mutations in the long genomic *Creb* construct in pBK-10, a 2.9-kb *EcoRV* fragment including exon 5 was subcloned into pBK for site-directed mutagenesis using PfuTurbo DNA polymerase (Agilent). To facilitate genotyping, a neighboring *StuI* site was removed by introducing a silent mutation at P132. Primers used: StuI/Y134F FOR: 5'-GGAAATCCTTTCAAGGAGGCCCTCCTTCAGGTATGTG; StuI/Y134F REV: 5'-CACATA CCTGAAGGAGGGCCTCCTTGAAAGGATTTCC. The mutations were confirmed by sequencing, and the mutant *EcoRV* fragment re-cloned into the pBK-10 to make pBK-10(Y134F). Exons 3-6 in the final 11.8-kb construct were confirmed by sequencing. 129/SvJae ES cells (from Kuo-Fen Lee) were cultured on irradiated primary neomycin-resistant MEF feeder cells on 0.2% gelatin-coated flasks in MEF medium containing LIF [3]. 20 x 10^6 cells were electroporated with 50 μg linearized pBK-10(Y134F) and plated onto MEF feeder cells. Cells were cultured in G418 (200 μg/mL) for 7-9 days or until G418-resistant ES cell colonies had grown to confluence. 400 colonies were picked under a microscope and transferred to 96-well plates containing MEF feeder cells. Clones were frozen down within one passage and expanded for Southern analysis [4]. Genomic DNA was digested *BstXI* and probed with a radiolabelled 945-bp *BamHI-EcoRI* fragment from pBK-7 corresponding to the *Creb* locus

immediately 3’ to the targeting cassette. Predicted wild-type (7.4-kb) and knock-in (8.7-kb) genomic fragments were detected (Figure S4). We used RT-PCR on total ES cell RNA to verify that CREB expression was unaffected. The Y134F mutation was confirmed by sequencing RT-PCR products. Two heterozygous Y134F ES clones were injected into blastocysts at the UCSD ES cell core facility. Chimeric founders were crossed to C57Bl/6 mice (Harlan). The floxed neo cassette was excised by crossing heterozygous agouti female pups to Protamine-Cre transgenic male mice on a mixed C57Bl/6-129Sv background (provided by Steven O’Gorman), which express Cre recombinase in the sperm, thus deleting loxP-flanked regions in early development [3]. Deletion of the neo cassette in $\textit{Creb}^{+/YF}\textit{Pro-Cre}^{+}$ offspring was confirmed by PCR. Following neo deletion, 100-bp comprising one loxP site and vector sequence remain, allowing discrimination between the wild-type and knock-in locus. $\textit{Creb}^{+/YF(\Delta neo)}$ offspring, hereafter referred to as $\textit{Creb}^{+/YF}$, were backcrossed to C57Bl/6 (Harlan) for 10 generations; the *Cre* transgene was not maintained.

Genotyping. For the YF strain: CREB_1F: 5’-CCTCAAAGCCCTTGCTTGCCATCACC; CREB_2F: 5’-CGTCGTGACCCATGGCGATGC; CREB-3R: 5’-GGCTATCATATAGCATCGCTGTGTCTG. $\textit{Creb}^{+}$ allele: 153-bp fragment, $\textit{Creb}^{YF\Delta neo}$ allele: 253-bp product. The *Dmd* genotype was determined by allele-specific PCR using Dyst-F 5’-AACTCATCAAATATGCGTGTTAGTG, Dyst-mdx-R 5’-GTCACT CAGATAGTTGAAGCCATTTAA, and Dyst-wt-R 5’-GTCACTCAGATAGTTGAAGCCATTTAG [5].

Breeding. Heterozygous mice were intercrossed to maintain the CREB-YF strain and to generate littermate or isogenic $\textit{Creb}^{+/+}$ and $\textit{Creb}^{YF/YF}$ mice for studies and breeding. To generate the large numbers of homozygous pups needed for primary cell isolation, generation-matched isogenic (after 10 generations of backcrossing) $\textit{Creb}^{+/+}$ and $\textit{Creb}^{YF/YF}$ homozygous male and female non-sibling mice were intercrossed.

Histological analysis. Skeletal muscles samples were flash frozen in liquid nitrogen-cooled isopentane and stored at -80˚C. 8-μm frozen sections were cut on a LeicaCM3050s cryostat and processed the same day.

REFERENCES

1. Sambrook J, Fritsch EF, Maniatis T, Cold Spring Harbor L (1989) Molecular cloning : a laboratory manual / J. Sambrook, E.F. Fritsch, T. Maniatis. Cold Spring Harbor, N.Y. :: Cold Spring Harbor Laboratory.
2. Canettieri G, Morantte I, Guzman E, Asahara H, Herzig S, et al. (2003) Attenuation of a phosphorylation-dependent activator by an HDAC-PP1 complex. Nat Struct Biol 10: 175-181.
3. O'Gorman S, Dagenais NA, Qian M, Marchuk Y (1997) Protamine-Cre recombinase transgenes efficiently recombine target sequences in the male germ line of mice, but not in embryonic stem cells. Proc Natl Acad Sci U S A 94: 14602-14607.
4. Brown T, Mackey K, Du T (2004) Analysis of RNA by northern and slot blot hybridization. Curr Protoc Mol Biol Chapter 4: Unit 4 9.
5. Amalfitano A, Chamberlain JS (1996) The mdx-amplification-resistant mutation system assay, a simple and rapid polymerase chain reaction-based detection of the mdx allele. Muscle Nerve 19: 1549-1553.
